# A Scoping Review of Rodent Studies Investigating the Epigenetic Mechanisms in the Brain Underlying the Effects of Diet on Depressive-like Behaviour

**DOI:** 10.3390/biomedicines10123213

**Published:** 2022-12-11

**Authors:** Carla L. Sánchez-Lafuente, Brady S. Reive, Lisa E. Kalynchuk, Hector J. Caruncho

**Affiliations:** Division of Medical Sciences, University of Victoria, Victoria, BC V8P 5C2, Canada

**Keywords:** diet, depression, animal models, brain epigenetics, DNA methylation, histone acetylation, microRNA

## Abstract

A healthy diet has been highly associated with a decreased risk for mental health problems such as major depression. Evidence from human studies shows that diet can influence mood but there is a poor understanding of the molecular mechanisms behind these effects, especially the role of epigenetic alterations in the brain. Our objective was to use the Preferred Reporting Items for Systematic Reviews and Meta-Analysis extension for Scoping Reviews (PRISMA-ScR) format to gather all recent studies using animal models that investigate direct or indirect (on the offspring) effects of diet on depressive symptoms, including studies that assess epigenetic mechanisms in the brain. In this format, two authors conducted independent database searches of PubMed, Web of Science, and Academic search premier using one search block “diet epigenetics depression” to find papers published between 2000 and 2022. Relevant studies were selected using pre-defined inclusion/exclusion criteria that were performed independently by the two authors before a subset of studies were selected for qualitative analysis. A total of 11 studies met the inclusion criteria for this systematic scoping review. We found that the literature focuses primarily on the effects of individual nutrients, instead of a specific diet, on despair-like behaviour and anxiety. Studies are heterogenous with the techniques used to asses epigenetic changes in the brain and therefore making it hard to reach common mechanistic explanations. However, all studies report diet-induced changes in the epigenome mainly by the action of DNA methylation, histone acetylation and microRNAs that are parallelel with changes in behaviour. Moreover studies show that inadequate maternal diets can make the offspring more susceptible to develop anxiety and depressive-like behaviour later in life, which is paralleled with changes in the epigenome. Overall, this systematic review shows that there is some literature suggesting a role of brain epigenetics on the diet-induced protective or detrimental effects, specifically on anxiety and depressive-like behaviour. However, studies are limited, lacking the study of some types of diets, behavioural tasks or epigenetic mechanisms. Nevertherless, it shows the importance of genome-environment interactions, bringing new insights towards mechanisms that could be involved in the pathophysiology of mood disorders as well as putative therapeutic targets.

## 1. Introduction

Major depressive disorder (thereinafter “depression”) is the leading cause of disability worldwide contributing to the overall global burden of disease [1,2]. The two main symptoms of depression are low mood and loss of pleasure (i.e., anhedonia), however, it often comes with a wide battery of other symptoms and comorbidities, the most common being anxiety. Depression is mainly characterized by an imbalance in the monoamine system, hence, conventional antidepressants that target the monoamine system are the gold-standard treatment for depression [3]. However, they have a therapeutic delay and do not work for a significant number of depression patients [4], suggesting that other mechanisms are also involved in the aetiology of this disorder. Alterations in neural plasticity as well as patterns of gene expression are also thought to be involved in depression since the NMDA receptor antagonist ketamine, has incredible fast-acting antidepressant properties that last for long [5,6]. Altogether, showing that depression is a complex and heterogeneous disorder, in which pathophysiology is not fully understood. 

Research in potential risk factors for depression is necessary to improve our understanding of the disorder and lead to the development of novel therapeutic targets or treatment approaches. A combination of genetic and environmental factors are thought to be responsible for the development of depression [7]. Environmental factors are external forces or stimuli that influences our body’s homeostasis, normal patterns of gene expression, neural circuit function, and ultimately behaviour. Common environmental factors include diet, exercise, weather, smoking, drugs or stress. Moreover, when a person or animal is exposed to certain environmental factors can be critical as exposure during developmental stages can have long-lasting effects shown later in adulthood as a consequence of epigenetic modifications [8]. Epigenetic modifications, refer to any inheritable changes that alter patterns of gene expression without altering the DNA sequence. The main epigenetic mechanisms known to date are; chromatin remodelling, histone modifications, DNA methylation or demethylation and non-coding RNAs. All these mechanisms are endogenously activated and necessary to maintain the proper function of the body, especially the brain. However exogenous inhibition or activation of these mechanisms by environmental factors can have undesired effects [9]. 

Over the past years, our understanding of epigenetic biology has coincided with an increased public interest in the impact of diet and lifestyle choices on health. A balanced diet or healthy diet has been associated with enhanced life expectancy and reduced risk for certain diseases, such as obesity, diabetes and cancer [10,11,12]. A healthy diet has also been associated with a decreased risk of developing depressive symptoms in humans [13]. Moreover, effects of diet can also be seen in the next generation as shown by inter-generational studies investigating the role of maternal diet on childhood mental health [14]. A large body of literature has shown associations between different types of diets (e.g., vegetarian, high-fat, hypercaloric, and Mediterranean diet) with alterations in mood and anxiety in humans [14]. However, there is little research focused on elucidating the mechanisms behind these effects. 

Mechanistic studies in the brain are very limited in humans but animal models can be used instead. There are many animal models for depression, mostly all developed in rodents, and they are based on genetics, environmental factors like stress or gene-environment interaction [reviewed in [15]]. Early life stress (ELS) can lead to dysregulation of the hypothalamic-pituitary-adrenal (HPA) axis which can manifest as depression [16]. Therefore, scientists have developed different ELS animal models to study the pathophysiology of depression [17]. A common method involves maternal separation, which has been proven to cause long-lasting effects on emotionality and stress responsiveness [18]. Moreover, stress has transgenerational effects that can be passed via epigenetic modifications into the offspring [19,20]. Diet is an example of an environmental factor that influences the physiological response to stress [21]. Each study included in this review involves manipulation of diet in animal models to investigate relationships between epigenetic modifications, diet and depression.

The goal of this review was to determine whether and which epigenetic mechanisms in the brain might underly the effects of diet on depressive-like behaviour. Because of the broad research question and limited literature available, we performed an exploratory scoping review following the Preferred Reporting Items for Systematic Reviews and Meta-Analysis extension for Scoping Reviews (PRISMA-ScR) format. We summarized and evaluated all the existing studies using animal models that looked at the effects of diet on depression symptoms and reported epigenetic alterations in the brain as part of the underlying mechanistic explanation for any observed behavioural changes. 

## 2. Methods

### 2.1. Search Strategy

We used three different databases to find the studies: PubMed, Web of Science and Academics Search Premier. In each one we did a search of “diet epigenetics depression” and all papers found were pooled into a folder in Mendeley. First, duplicates were removed, then with the help of the search tool looking for “review” we removed all the review papers before reading the titles and abstracts of the remaining papers and removing those that were non-related to the topic or did not use rodent animal models. We decided to exclude human studies due to limitations pertaining to methodologies available for elucidating molecular mechanisms in the brain. After an initial screening the remaining studies were screened in their entirety and those that passed the inclusion-exclusion criteria were included in our final selection. 

### 2.2. Eligibility Criteria

Articles were eligible for inclusion if they: (i) were full-text journal articles; (ii) in English; (iii) contained diet, depression and epigenetics in their abstract; (iv) comprised in rat or mice study; (v) included molecular or cellular data on epigenetic mechanisms; (vi) a cohort or case-control study. Studies were excluded if they: (i) were published in languages other than English; (ii) were published before 2000; (iii) were review papers; (iv) behaviour only papers; (v) mechanistic studies only; (vi) no relation to depressive symptoms, diet or epigenetics; (vii) preliminary data papers; (viii) study in humans.

### 2.3. Extraction and Synthesis of Data

A detailed examination of the studies was carried out to evaluate the strength of the evidence and the validity of its inclusion in this review. Data extraction and compilation tables were developed only by one researcher with the following information: publication characteristics (authors, publication year and journal); characteristics of the experimental model (animal strain, age, regions studied, depression model) and main characteristics of the intervention (diet, depressive model and epigenetic measures). 

### 2.4. Risk of Bias

The risk of bias was assessed using the SYRCLE tool (Systematic Review Centre for Laboratory animal Experimentation) (Nijmegen, The Netherlands). This instrument looks at biased aspects that play a specific role in animal intervention studies whose goal is to establish a consistent evaluation of the methodological quality in the field of animal experimentation. In order to increase transparency and reproducibility, different signalling has been formulated in the following areas: (1) random sequence generation, (2) baseline characteristics, (3) allocation concealment, (4) random housing, (5) investigator blinding, (6) random outcome assessment, (7) blinding of outcome data, (8) incomplete outcome data, (9) selective outcome reporting, and (10) ethical considerations.

## 3. Results

This section describes in detail the process of literature selection, quality ratings of the studies, methodology used and also a summary of the results of these studies.

### 3.1. Selection

**Figure 1:**
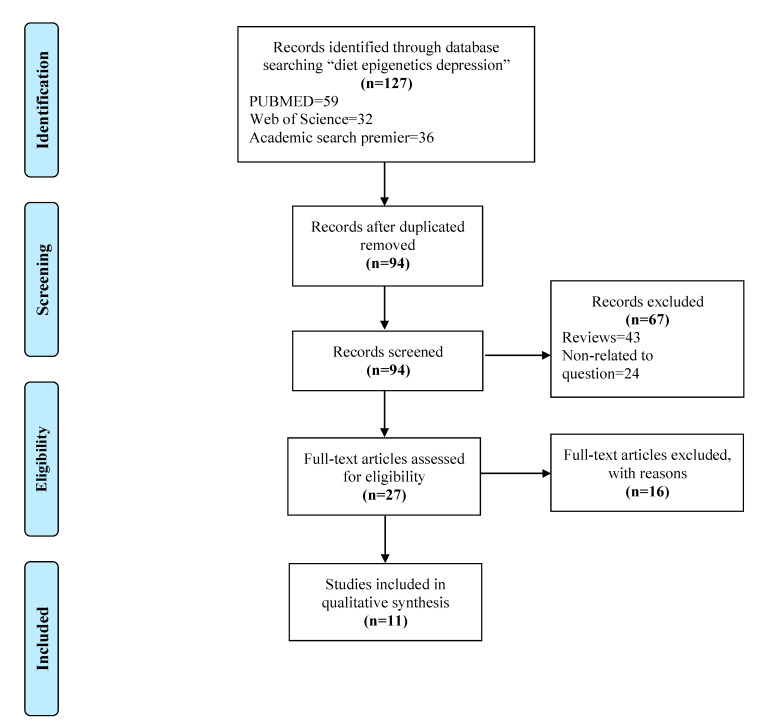
PRISMA flow diagram followed to get to the final selection of studies included in the review.

A total of 127 studies were identified in the initial search, after duplicates were removed 94 studies were left for screening. The initial screening excluded 43 reviews and 24 studies that did not relate to the question, leaving 27 studies for the final screening. In total, 11 studies met the inclusion criteria for qualitative analysis. This whole selection process was performed by two reviewers that reached consensus. Complete details of screening, filtering and our selection process for studies included in this review are shown in Figure 1.

### 3.2. Study Characteristics

The general characteristics of the final selection are summarized in Table 1. All are preclinical studies conducted in rodents, five in mice and five in rats. No other experimental studies without the use of animals were identified and human studies were not chosen to be included in this review. All studies have been published in peer reviewed journals of different focuses, from behavioural to molecular. Most studies included both sexes in the studies and half looked at the effects of maternal diet on the offspring at different stages of life (fetus to adult) while the other half looked at the direct effects of diet on adult rodents. 

### 3.3. Study Quality and Risk of Bias

The results of our risk of bias assessment of the 11 studies included in this systematic review are shown in Table 2. We assessed the level of bias by low risk, unclear and high risk. When it was unclear was mostly due to missing details, but if it was something crucial in the experiment, we considered a high risk of bias. All studies included here provided a good description of random sequence generation as well as baseline characteristics, however some studies provided more specific details about the diet involved than others, especially related to the composition of control diets. Moreover, most studies included in this review did not report information pertaining to investigator blinding or random outcome assessment. Most studies did not provide a clear description of allocation concealment, referring to the randomization of which subject receives which treatment. Risk of bias on selective outcome reporting was considered when there were missing figures to support specific results reported or were missing discussion of some results relevant to the topic of this review. Overall, we consider that all included studies reached a desired quality for the purpose of this review, yet future studies should improve their reporting by making it more clear and reduce bias which will allow future systematic reviews get to reliable specific conclusions.

### 3.4. Study of Diet

Each study used a different dietary approach studying different aspects of the diet; (1) macronutrient content, (2) micronutrient content, (3) calorie intake, and (4) methyl donor intake. Of the articles included, four studies looked at the effects of dietary macronutrients, two studied micronutrients both supplementation or deficiency effects, two studies manipulated calorie intake instead of a specific dietary component and the remaining two investigated the effects of a methyl donor rich or deficient diet. Moreover, four studies look at the effects of maternal diet on the offspring and six studies look at the direct effects of diet on the adult rodent, see summary in Table 3.

Lipids, proteins and carbohydrates are the macronutrient components of the diet that provide most of the caloric energy. Studies used commercial food pellets containing specific amounts of these macronutrients to feed the rodents whereas control groups were fed a control or standard diet. The amount of detail pertaining to specific macronutrient content in the diet is variable across studies, however each describes a 65% carbohydrate content. The percentage of fat and protein varies between studies. Three studies used high-fat diets with the same percentage of fat at 60% [26,30,32]. Three studies used high-carbohydrate diets, in general, they are quite similar diets including 70% carbohydrates [25], 78% carbohydrates rich in sucrose [30] or 65% fructose [23]. Finally, most studies use regular levels of protein, around 15–20% protein content, and one study uses a low-protein diet consisting of only 8% of protein [25]. On the other hand, we have vitamins and minerals which are considered micronutrients. Two studies focus on manipulating vitamins B12, B6, and folate, which are important micronutrients for the body. Both studies look at the effect of a diet deficient in specific vitamins [28,31]. Similarly, two studies investigated the effects of calories in the diet, including a hypercaloric diet or a calorie-restricted diet [22,27]. Lastly, two studies investigated the effects of supplementation or depletion of methyl donors in the diet such as choline [24,29]. 

### 3.5. Behavioural Measures

All studies measure mood-related behaviour with a battery of behavioural tests designed for rodents (Table 3). The main depression-like behaviours measured were despair-like behaviour and anhedonia. Several studies also assessed anxiety-like behaviour and social interaction. Although anxiety is not a core symptom of depression, we included papers that only assessed anxiety as there exists high comorbidity with depression [33].

The most common behavioural test for measuring despair-like behaviour is the forced swim test (FST), which has been validated in animal models for depression for measuring despair-like behaviour in rats or the tail suspension test (TS) for mice. All studies in this systematic review conducted the FST, one conducted TS and one conducted neither test (assesses anxiety only). To evaluate anhedonic behaviour, the sucrose preference test (SPT) is the most commonly used. Only four studies looked at anhedonia and each used this test. Anxiety can be assessed with several tests like the open field test (OFT), the elevated plus maze test (EPM) or variations of it like the elevated zero maze test (EZM). Most studies included here use a combination of two of these tests. Two studies applied the light/dark box test (BT) to assess anxiety-like behaviour. Finally, for social interaction, there is the social interaction test or task (SI), and although parameters are variable between research groups, they all assess how the subject interacts with a novel, unfamiliar mouse or rat. A total of four studies included this test in their behavioural repertoire. Other behaviours were also assessed in several studies presented in this review, however, they will not be discussed as they do not fall under the topic of this review. 

### 3.6. Epigenetic Mechanisms

The two main epigenetic modifications assessed in these studies were the methylation of the DNA and histone modifications (Table 3). One study evaluated microRNAs (miRNAs) and another evaluated changes to gene expression profile. A broad range of biochemical techniques were employed to evalaute various regions of the brain including the hypothalamus [22,26], the cortex [27,28,30,31], the hippocampus [29,31,32], the amygdala [24,25], the striatum [23,31,32], nucleus accumbens, ventral tegmental area [31] and subventricular zone [27]. Despite some regions being more relevant to depression than others, they are related to each study paradigm.

### 3.7. Effects of Diet on Mood

All studies reported changes in different behaviours. Depression is highly comorbid with anxiety, therefore in this section we included and will discuss the findings on the effects of diet on both depression and anxiety-like behaviours. 

Macronutrient content effects on mood: A 60% fat diet showed direct effects on mood, increasing despair-like behaviour in adult rats without altering anxiety-like behaviour in the EPM compared to controls [26]. A maternal high-fat diet and a mixed diet with high-fat and a perinatal low-protein diet all produced despair-like behaviour in the offspring [25]. On the other hand, a maternal high-fat diet reduced anxiety-like behaviour in the offspring, measured in the EPM, but consistently increases immobility in the FST in both sexes [30,32]. Male, but not female, offspring of low-protein diet-fed mothers displayed anxiety behaviours under acute stress [25]. A high-fructose diet caused increased anxiety compared to normal chow-fed rats [23] and a maternal high-carbohydrate diet produced despair-like behaviour and increased anxiety-like behaviour in the offspring compared to controls [30]. 

Micronutrient content effects on mood: A vitamin-deficient diet lacking vitamin B6, B9, and vitamin B12 increased despair-like behaviour in adult mice while diets with individual vitamin deficiencies did not affect despair-like behaviour [31]. Interestingly, B6 deficiency caused increased anhedonia in the SPT while moderate vitamin B12 deficiency did not bring about any difference in anhedonia compared to the control group [28,31]. However, severe vitamin B12 deficiency led to a significant decrease in sucrose preference in the female mice as compared to control and mice moderately deficient in B12 [31]. A diet lacking vitamin B6, B9, or B12 either individually or combined caused no anxiety [31]. Elsewhere, vitamin B12 restricted diet was shown to increase anxiety-like behaviour relative to normal chow-fed animals without affecting social interaction behaviour in mice [28]. However, in the study by Xu and colleagues, alterations were observed to time interacting with stranger mice in the social interaction test in B6, B9, and B12 deficient groups [31].

Calorie intake effects on mood: Calorie restriction dramatically reduced depression-like behaviour in male and female mice, with increased latency to immobility and reduced total immobility during the FST. In striking contrast, both male and female mice deficient in orexin, a neuropeptide that regulates appetite and wakefulness, did not show a calorie restriction-induced improvement in the forced swim test [22]. Moreover, calorie restriction increased social interaction in control mice and completely reversed social interaction deficits induced by social defeat stress [22]. Adult offspring of dams fed a hypercaloric diet enriched with omega-6 polyunsaturated fatty acids (ω-6 PUFA) showed more anxiety-like behaviour than those from dams fed a standard diet [27].

Methyl donor intake effects on mood: Dietary methyl donor depletion induced depression-like behaviour in rats bred for low behavioural response to novelty, but not anxiety-like behaviour, whereas methyl donor supplementation had an anxiolytic effect in the EPM and significantly reduced immobility in the FST relative to controls [24]. Although methyl donor-supplemented animals initiated social interaction more quickly, no significant change was observed to time in close proximity to the social stimuli compared to methyl donor deprived and control groups [24]. Moreover, maternal citicoline supplementation decreased the depressive-like behaviour induced by perinatal asphyxia in the offspring [29].

### 3.8. Epigenetic Mechanisms behind the Effects of Diet on Mood

Studies evaluating epigenetic mechanisms in their approach were included in this systematic review with the goal to evaluate the relationship between epigenome changes and the effects of diet on mood, specially depressive-like behaviours and anxiety. Interestingly, most studies show brain region-specific changes in DNA methylation caused by diet, affecting various transcription pathways. For example, a hypercaloric maternal diet enriched with ω-6 PUFA caused DNA methylation and chromatin accessibility profile changes in the endocannabinoid system in the subventricular zone, an important site of neurogenesis and neural proliferation, and cortex of fetuses and increased depression-like behaviours [27]. An obesity-inducing diet of 60% fat content, which produced a depressive phenotype, increased global 5-methylcytosine (5mC) levels and decreased 5-hmc levels compared to controls, although no significant changes to DNA methyl transferases or pyrosequencing were observed [26]. A similar diet provided during pregnancy and lactation decreased the methylation of CpG islands at the Cnr1 promoter in the hippocampus of both adolescent and adult offspring of the dams fed with the diet [32]. As predicted, a methyl donor diet increased global DNA methylation, increasing 5-mC levels compared to rats receiving a regular diet or a methyl donor-depleted diet which correlated with a reduction of depression-like behaviour [24]. Perinatal low-protein diet (LPD) increased basal protein levels of the Early Growth Response 1 (EGR1) in the amygdala. It also caused an increased methylation of the EGR1 binding sites in the neuropeptide Y 1 receptor gene (Npy1r) of females compared to controls [25]. These sex specific epigenetic alterations paralleled behavioural consequences of perinatal LPD as males showed increased depression-like and anxiety-like behaviour under acute stress, whereas females showed a reduction of immobiliy [25]. 

Histone posttranslational modifications including methylation or acetylation were also reported in the articles reviewed. As previously mentioned, dietary deficiency of certain B vitamins increased depression-like behaviour, and interestingly, these animals also presented with decreased numbers of H3K9me2 positive cells in the CA3 area of the hippocampus [31]. Moreover, in agreement with previous studies, Ghosh and colleagues found that mice severely deficient in B12 exhibit a depressive phenotype and increased global expression of HDAC4, a histone deacetylase enzyme [28]. Molecular studies showed that high-fat diet leads to persistent transcriptomic alterations [32]. A high-fructose diet was shown to increase anxiety-like behaviour and decrease levels of two histone deacetylase enzymes, sirtuin 1 and 7 [23]. 

Finally, non-coding RNA also plays a role in modulating the epigenome. A study found that maternal citicoline-supplemented diet, a methyl donor, upregulated miR124 and downregulated miR132 and miR134 in the hippocampus [29]. High-fat diet was also shown to affect miRNA expression levels in the adolescent and adult male offspring, increasing miR-212-5p in the cortex and striatum and miR-154-3p was increased in the striatum but decreased in the hippocampus [32]. 

## 4. Discussion

In the present report, we summarized the existing literature investigating the effects of diet on mood in rodent animal models where epigenetic mechanisms in the brain were assessed. In summary, we found a limited and heterogenous body of studies that studied either the direct or indirect (transgenerational) effects of diet. In general, studies reported that diet affected depression-like behaviour, which was associated with various epigenetic modifications in the brain, including DNA methylation, histone acetylation and the action of miRNAs. 

With the increased global consumption of processed and fast foods, there is a raised interest in elucidating potential links between unbalanced diets containing disproportionate amounts of fats, carbohydrates and proteins and susceptibility to mood disorders like depression and anxiety. In this literature review, we found evidence supporting that a high-fat diet causes increased despair-like behaviour in the FST, although it also reduced anxiety in the EPM. Interestingly both studies that reported these effects also showed that these animals had increased global 5-mC and decreased 5-hmc levels as well as persistent alterations in transcriptomic markers compared to controls. These are not the only studies in the literature reporting effects of diet in DNA methylation patterns [34], but they are the only, at least that we found, reporting a link with mood alterations. Additionally, one study also found a decrease in MiR-154-3p and MiR-212-5p in the hippocampus. MicroRNAs play important roles in the development and progression of depression through the regulation of protein expression [reviewed in [35]]. MiR-154-3p has been observed to be important for neuronal function, synaptic plasticity, memory formation and blood-brain barrier integrity [36] and MiR-212-5p influences apoptosis, inflammation, and cytotoxicity [37]. Overall, results suggest that a high-fat diet could influence depression-like behaviour via the alteration of miRNAs and DNA methylation, potentially mediated by fatty acids [38]. 

A low-protein diet caused increased despair-like behaviour but decreased anxiety-like behaviour in male offspring only. One study suggested DNA methylation is one mechanism responsible for the sexually dimorphic effects of diet on anxiety-like behaviour. Females showed increased methylation on the early growth response 1 (EGR1) binding sites in the *Npy1r* gene when exposed to a low-protein diet compared to controls. Two studies concluded high-carbohydrate content in diet induces anxiety-like behaviour. Molecular data from one study showed that a high-carbohydrate diet decreases two histone deacetylase enzymes, sirtuin 1 and 7, which supports the hypothesis that diet-induced alterations in mood could be mediated by epigenetic changes in the brain. 

Other important components of the diet are micronutrients, which includes vitamins and minerals. Multivitamin supplementation in humans has been shown to reduce negative mood states, including depression, anxiety, and stress [39,40]. In this review only the effects of some vitamins were studied. Vitamin B12 deficiency increased anhedonia and anxiety-like behaviour, and a combined deficiency of vitamin B12, B6 and B9 increased despair-like behaviour. Previous studies have associated decreased levels of histone H3 lysine 9 di-methylation with potentiated depressive-like behaviours in mice [41], and interestingly, vitamin B12 deficiency caused the same effect. Moreover, B12 deficient animals presented high histone deacetylase 4 levels, which is typically associated with gene repression [42]. Vitamins B6, B9, and B12 are the most prominent B vitamins involved in homocysteine metabolism that provides a substrate for genomic and non-genomic methylation [43]. Here we show that several studies report an association between B12 and depression symptomatology that potentially occurs through histone modifications and other epigenetic changes. 

Chronic calorie restriction effects on depression and anxiety-like behaviours have also been well studied [44,45], however, an underlying neurobiological mechanism involving epigenetic modifications has only recently been considered as potentially responsible for these relationships. A few studies included in this review found that calorie restriction had a strong effect on social behaviour, increasing baseline levels and reversing the effects of social defeat stress. It also caused a dramatic antidepressant-like effect in both sexes, which seemed to be related to epigenetic modifications that influence activation of orexin neurons. Only one study specifically studied the effects of a hypercaloric diet on mood, and although they observed an increase in depression-like behaviour and anxiety-like behaviour, the study used a diet enriched with ω-6 PUFAs to study the effects of modulating the endocannabinoid system rather than the impact of calories in the diet on mood. As one would have expected, a methyl donor diet increased global DNA methylation, increasing 5-mC levels compared to rats receiving a regular diet or a methyl donor-depleted diet. Interestingly, increased methylation was correlated with improvement in depression-like symptoms. Dietary methyl donor depletion worsened depressive symptoms and anxiety, whereas methyl donor supplementation improved both depression- and anxiety-like behaviour. As previously mentioned, non-coding RNAs also play a role in modulating the epigenome [35] and data from one study showed upregulated miR124 and downregulated miR132 and miR134 in the hippocampus after a maternal citicoline (methyl donor) supplemented diet. Several targets for miR-132 have been described, including mediators of neurological development and synaptic transmission [46,47]. Additionally, miR-134 plays a prominent role in development, and altered levels have been observed in individuals diagnosed with both schizophrenia and bipolar disorder [48,49].

Although there were varied methodological approaches in the studies included in this review, some commonalities were observed with similar rodent strains and behavioural tests. On the other hand, there was a wide range of diets included, but each type of dietary approach was at least studied by two different studies. In terms of sex, studies that looked at the transgenerational effects of diet included both sexes, otherwise studies were conducted predominantly in males. Relationships between diet and a depressive phenotype are observed in both sexes. On the other hand, a wide variation was found for the age of the animals used in the experiments. Despite most studies investigating the direct effects of diet using adult rodents of 7 or more weeks of age, offspring studies ranged from investigating the early effects of diet on fetuses to long-term effects on 8-week-old adults. This makes it harder to compare results between studies, however, it provides useful information for a wider population range. All studies conducted their neurochemical analyses on brain samples but knowing that the brain communicates with the gut and vice versa [50], one would have expected some studies also looking at the gut or peripheral blood too. Finally, most studies showed an adequate quality, with low risk of bias. However, a few studies were missing a lot of details which we reported as unclear bias and for future systematic reviews on this topic, this limitation should be considered when reporting specific results or reaching any conclusions. 

All in all, despite these limitations, this systematic scoping review shows that diet-induced effects on depressive-like behaviour and anxiety might be mediated by epigenetic mechanisms in the rodent brain, but the amount of research found is very heterogenous and limited. Most studies focus primarily on individual nutrients instead of an overall diet approach, hence we suggest that future research is conducted studying other specific diets that humans consume regularly (e.g., plant based, keto). Moreover, we would like to see further effects of also at the level of the peripheral nervous system as well as the gut and include a wider battery of depressive-like behavioural tests. We hope that this review serves as a base for future studies to be conducted on this field, taking into account all the limitations and identified gaps, so they can expand the current knowledge. Learning more about the molecular mechanisms underlying the beneficial or detrimental effects of diet will potentially allow us to manipulate it and use it as a therapeutic tool for the treatment of mood disorders.

## Figures and Tables

**Table 1:** General characteristics of included studies.

Studies	Journal Name	Peer Reviewed	Subject of Study (for Diet)	Age (Start Experiment)	Sex
Lutter et al., 2008 [22]	Journal of Neuroscience	Yes	Orexin-deficient C57BL/6 mice	8–10 weeks old	Male and female
Reddy et al., 2016 [23]	Journal of Biosciences	Yes	Sprague–Dawley rats	7 to 8 weeks old	Male
McCoy et al., 2017 [24]	Behavioural Brain Research	Yes	Sprague Dawley rats HR/LR bred line	adult (do not specify age)	Male and female
Nätt et al., 2017 [25]	Scientific Reports	Yes	Sv129 mice and C57BL/6 J(JAX)mice for mechanistic study	Offspring (10 weeks old)	Male and female
Cui et al., 2018 [26]	Experimental Physiology	Yes	Wistar rats	21 days old	Female
Cinquina et al., 2020 [27]	Molecular Psychiatry	Yes	C57Bl6/J, cholecystokinin (CCK)BAC/DsRed and CCKBAC/DsRed::GAD67gfp/+ transgenic mice	E18.5 fetuses	Male and female
Ghosh et al., 2020 [28]	Nutritional Neuroscience	Yes	C57BL/6 mice	12 weeks old	Female
Isac et al., 2020 [29]	Neonatology	Yes	Wistar rats	Offspring (6 day old)	Male and female
Gawlińska et al., 2021 [30]	Developmental Cognitive Neuroscience	Yes	Wistar rats	Offspring (28 days old)	Male and female
Xu et al., 2021 [31]	Psychopharmacology	Yes	C57BL/6 J mice	Offspring (8 weeks old)	Male and female
Gawliński et al., 2021 [32]	Nutrients (MDPI)	Yes	Wistar rats	Offspring (4 and 8 weeks old)	Male and female

**Table 2:** Risk of bias assessment.

Studies	Random Sequence Generation (Selection Bias)	Baseline Characteristics (Selection Bias)	Allocation Concealment (Selection Bias)	Random Housing (Performance Bias)	Investigator Blinding (Performance Bias)	Random Outcome Assessment (Detection Bias)	Blinding of Outcome Data (Detection Bias)	Incomplete Outcome Data (Attrition Bias)	Selective Outcome Reporting (Reporting Bias)
Lutter et al., 2008 [22]									
Reddy et al., 2016 [23]									
McCoy et al., 2017 [24]									
Nätt et al., 2017 [25]									
Cui et al., 2018 [26]									
Cinquina et al., 2020 [27]									
Ghosh et al., 2020 [28]									
Isac et al., 2020 [29]									
Gawlińska et al., 2021 [30]									
Xu et al., 2021 [31]									
Gawliński et al., 2021 [32]									

Color code: white = low risk of bias, gray = unclear risk of bias and black = high risk of bias.

**Table 3:** Diet, behaviour and epigenetic characteristics of included studies.

Studies	Diet	Diet Characteristics	Animal Model	Behavioural Test	Epigenetic Mechanism	Epigenetic Measures
Lutter et al., 2008 [22]	Calorie restricted diet	Food pellet equal to 60% of their average daily food intake before onset of the dark phase (7:00 P.M.)	Chronic social defeat stress	FST, SI	Functional histone alteration	Dimethyl lysine 9 H3 (H3K9me2) at orexin promoter
Reddy et al., 2016 [23]	High-fructose diet	Control diet (65% corn starch diet), high-fructose diet (65% fructose diet), resveratrol group (65% fructose diet with 10 mg/kg/day of resveratrol) and metformin group (65% fructose diet with300 mg/kg/day of metformin)	Diet-induced prediabetic condition causing depressive phenotype	OFT, BT	Histone deacetylation	Levels of NAD-dependent class III histone deacetylases (sirtuins)
McCoy et al., 2017 [24]	Methyl donor rich or depleted diet	Control diet (semisynthetic L-amino acid-complete rodent diet), methyl donors depleted diet (L-amino acid-defined rodent diet lacking 90% of normal requirements of choline, folate, and methionine), methyl donors supplemented diet (chow fortified with increased amounts of folic acid, choline, methionine, and Vitamin B12)	Model of temperamental differences, Low Novelty Responder (LR) and High Novelty Responder (HR) rats.	EPM, OFT, SI, and FST	DNA methylation	DNA methyltransferase (DNMT) expression, global DNA methylation (5-methylcytosine) and methylome sequencing
Nätt et al., 2017 [25]	Maternal low protein diet	Low protein diet with 8% protein content (78% carbohydrates; 5% fat) or regular chow with 20% protein content (66% carbohydrates; 5% fat)	Early life stress by maternal low protein diet	EPM, FST, OFT,	Interplay between EGR1 and epigenetic mechanisms like DNA methylation	Transcription of the Egr family genes, DNA-methylation in Npy1r, Interaction of Npy, Npy1/2/5r with EGR1
Cui et al., 2018 [26]	DIO-diet induced obesity model	DIO-60% fat diet for 3 months/control has normal rodent chow	Diet induced obesity	SPT, FST, OFT, EPM	DNA methylation	DNMTs levels, DNA methylation
Cinquina et al., 2020 [27]	Maternal ‘Western’ diet	Hypercaloric diet enriched ~15-fold in ω-6 PUFAs while maintaining the ω-3:ω-6 PUFA ratio at 1:8 as in standard chow or to a standard diet before and during pregnancy	Early life stress by maternal hypercaloric enriched diet	OFT, EPM	DNA methylation and chromatin accessibility	Chromatin accessibility of transcription factors and DNA methylation
Ghosh et al., 2020 [28]	Vitamin B12 deficient diet	AIN76A control diet with cellulose, AIN-76A diet deficient in vitamin B12 with pectin. Vitamin B12 content in the restricted diets 62.5% lesser than the control diet	Chronic B12 deficiency before and during pregnancy	OFT, EPM, BT, SPT, SI, TS	Histone modifications	Gene expression of class I, class IIa and class IIb HDACs, histone methyltransferases
Isac et al., 2020 [29]	Maternal citicoline-supplemented diet	Citicoline 200 mg/kg body weight/day in water (CDS) or standard diet	Perinatal asphyxia	OFT, FST	MicroRNAs	MicroRNA expression levels
Gawlińska et al., 2021 [30]	High fat diet, high carbohydrate diet and mixed diet	Standard diet (SD; 65% carbohydrate, 13% fat, 22% protein, 3.4 kcal/g), high-fat diet (HFD; 60% fat, 5.31 kcal/g), high-carbohydrate diet (HCD; 70% carbohydrate: rich in sucrose—40%, 3.77 kcal/g) or mixed diet (MD; 56% carbohydrate, 28% fat, 3.90 kcal/g)	Modified maternal diet	EZM, FST, SPT	Epigenetic profile changes	Gene expression by RNA sequencing (they don’t really look at a specific mechanism)
Xu et al., 2021 [31]	Vitamin B6, B9, B12, and folate deficient diets	Control diet (0.07% vitamin B6, 0.02% folate, 0.25% vitamin B12, with an energy content of 17.9% protein, 7.0% fat, and 64.4% carbohydrate) (NOR); vitamin B6 deficient diet (no B6) (DB6); folate-deficient diet (no folate) (DB9); vitamin B12–deficient diet (no B12) (DB12); and vitamin B6–, B9–, and B12–deficient diet (no B6, folate, and B12) (DB6912)	Early life stress by maternal vitamin deficient diet	SPT, OFT, FST, TST, SI	Functional histone alteration	Dimethylated lysine 9 on histone H3 (H3K9me2)
Gawliński et al., 2021 [32]	Standard diet and high fat diet	Standard diet (13% fat, 3.4 kcal/g) or High-fat diet (60% fat, 5.31 kcal/g)	Modified maternal diet	FST	DNA methylation and alterations in miRNA	CpG methylation (Cnr1 Promoter) and expression levels of miR-154-3p and miR-212-5p

Abbreviations: Elevated Plus Maze (EPM), Elevated Zero MAZE(EZM), Open Field Test (OFT), Social Interaction Test (SI), and Forced Swim Test (FST). Light/dark box test (BT), Sucrose Preference Test (SPT), Tail Suspension test (TS).

## Data Availability

Not applicable.

## Abbreviations

Deoxyribonucleic acid (DNA), Ribonucleic acid (RNA), 5-methylcytosine (5-mC), 5-hydroxymethylcytosine (5-hmc), Histone deacetylase (HDAC), Elevated Plus Maze (EPM), Elevated Zero Maze (EZM), Open Field Test (OFT), Social Interaction Test (SI), and Forced Swim Test (FST). Light/dark box test (BT), Sucrose Preference Test (SPT), Tail Suspension test (TS), MicroRNAs (miRNAs), Systematic Review Centre for Laboratory animal Experimentation (SYRCLE), Early Growth Response 1 (EGR1), Low-protein diet (LPD).
